# Semaglutide in cognitive dysfunction: neuroprotective potential, clinical trial limitations, and a prevention-focused framework

**DOI:** 10.3389/fnagi.2026.1851072

**Published:** 2026-06-05

**Authors:** Ahmad H. Alhowail, Abdulaziz K. Al Mouslem, Mohammed A. Almatrafi, Maha A. Aldubayan

**Affiliations:** 1 Department of Pharmacology and Toxicology, College of Pharmacy, Qassim University, Buraydah, Saudi Arabia; 2 Department of Pharmaceutical Sciences, College of Clinical Pharmacy, King Faisal University, Al Ahsa, Saudi Arabia

**Keywords:** Alzheimer’s disease, clinical trial, cognitive dysfunction, GLP-1 receptor agonist, neuroinflammation, neuroprotection, semaglutide, vascular dementia

## Abstract

Metabolic dysfunction is increasingly recognized as a pivotal factor in cognitive decline and neurodegenerative diseases, such as Alzheimer’s disease (AD). Glucagon-like peptide-1 receptor agonists (GLP-1RAs), notably the long-acting agonist semaglutide, exhibit significant metabolic efficacy and pronounced neuroprotective effects across a broad spectrum of preclinical models. This is corroborated by extensive epidemiological studies that consistently link GLP-1RA use with a decreased incidence of dementia. Nevertheless, promising preclinical and observational findings have not been mirrored in clinical success for the treatment of established AD. Recent negative outcomes from the pivotal phase 3 EVOKE and EVOKE+ trials, which demonstrated no clinical benefit of oral semaglutide in patients with early AD, have resulted in a notable translational paradox. This review critically examines the mechanistic, preclinical, epidemiological, and clinical evidence concerning the impact of semaglutide on cognitive function to reconcile these conflicting findings. Preclinical studies have revealed complex neuroprotective mechanisms, including suppression of neuroinflammation, restoration of metabolic function, and activation of pro-survival pathways. Conversely, clinical trials in symptomatic AD have been unsuccessful, although modest and clinically insignificant changes in cerebrospinal fluid biomarker levels have been observed. We propose the hypothesis that the current body of evidence is consistent with a prevention-focused model, wherein semaglutide’s primary value may lie in modifying the upstream metabolic and inflammatory drivers of neurodegeneration, such as those prevalent in vascular and metabolic cognitive impairment, rather than reversing established amyloid-driven AD pathology. This hypothesis, however, remains speculative and requires prospective validation in appropriately designed trials. This review seeks to resolve the apparent contradictions in the literature and propose future research directions centered on appropriate patient populations and therapeutic windows.

## Graphical Abstract

**Figure:**
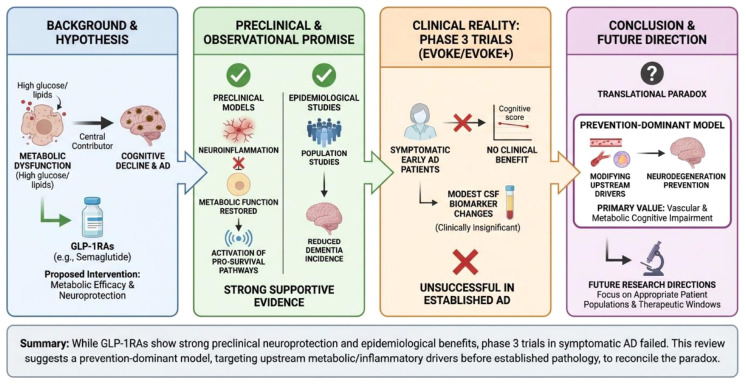
Semaglutide may reduce cognitive dysfunction by improving metabolic control and inflammation, but current evidence supports a stronger role in early prevention than in treating established Alzheimer’s disease.

## 1 Introduction

The pursuit of a disease-modifying therapy for Alzheimer’s disease (AD) has extended over several decades, resulting in numerous unsuccessful clinical trials in this field. The recent conclusion of major phase 3 trials for the oral glucagon-like peptide-1 (GLP-1) receptor agonist semaglutide represents another notable setback in this endeavor (Scragg et al., 2025). However, this outcome highlights a significant translational paradox. Semaglutide, a highly efficacious metabolic drug, has consistently exhibited substantial neuroprotective effects across a diverse array of preclinical models, addressing the key pathological features of neurodegeneration, including neuroinflammation, metabolic dysfunction, and apoptosis (Tipa et al., 2024). Moreover, large-scale epidemiological studies have consistently associated GLP-1 receptor agonist use with a significantly reduced risk of developing dementia (Lin et al., 2025; Wang et al., 2024).

The observed discrepancy between promising preclinical and epidemiological data and the negative outcomes of a large-scale clinical trial in established Alzheimer’s disease (AD) necessitates a thorough analysis. As the global population ages, the prevalence of cognitive dysfunction is increasing worldwide, highlighting the urgent need for effective therapeutic and preventive strategies (Scheltens et al., 2021). The well-documented association between metabolic conditions, such as type 2 diabetes mellitus (T2DM) and obesity, and the risk of dementia has created opportunities for repurposing metabolic drugs for neurological disorders (Long and Holtzman, 2019). The characterization of AD as “Type 3 diabetes,” a condition of brain-specific insulin resistance, has directed attention toward GLP-1 receptor agonists as potential neuroprotective agents (de la Monte and Wands, 2008).

This review aims to provide a thorough and critical assessment of the current understanding of semaglutide’s role in cognitive dysfunction. We will integrate evidence ranging from fundamental pharmacology and molecular mechanisms to divergent findings from epidemiological studies and large-scale clinical trials. By striving to reconcile the disparity between its considerable neuroprotective potential and recent clinical challenges, we can enhance our understanding of the potential role of semaglutide and other GLP-1 receptor agonists in combating cognitive decline.

Recent evidence suggests that GLP-1 receptor agonists may offer their most significant neurological benefits during the preclinical phases of neurodegeneration by influencing upstream metabolic and inflammatory risk factors rather than reversing established pathology. Consequently, this review examines the hypothesis that semaglutide might primarily serve as a prevention-oriented neuroprotective intervention rather than a disease-modifying therapy for symptomatic Alzheimer’s disease. However, this review also critically evaluates alternative explanations for the translational gap, including limited central nervous system penetration, insufficient pharmacodynamic target engagement, a mismatch between the drug’s metabolic mechanisms and the amyloid-driven pathology of established AD, and the inherent limitations of preclinical models. A balanced appraisal of all these hypotheses is essential to draw appropriate conclusions from the available evidence.

## 2 Methodology

**FIGURE 1:**
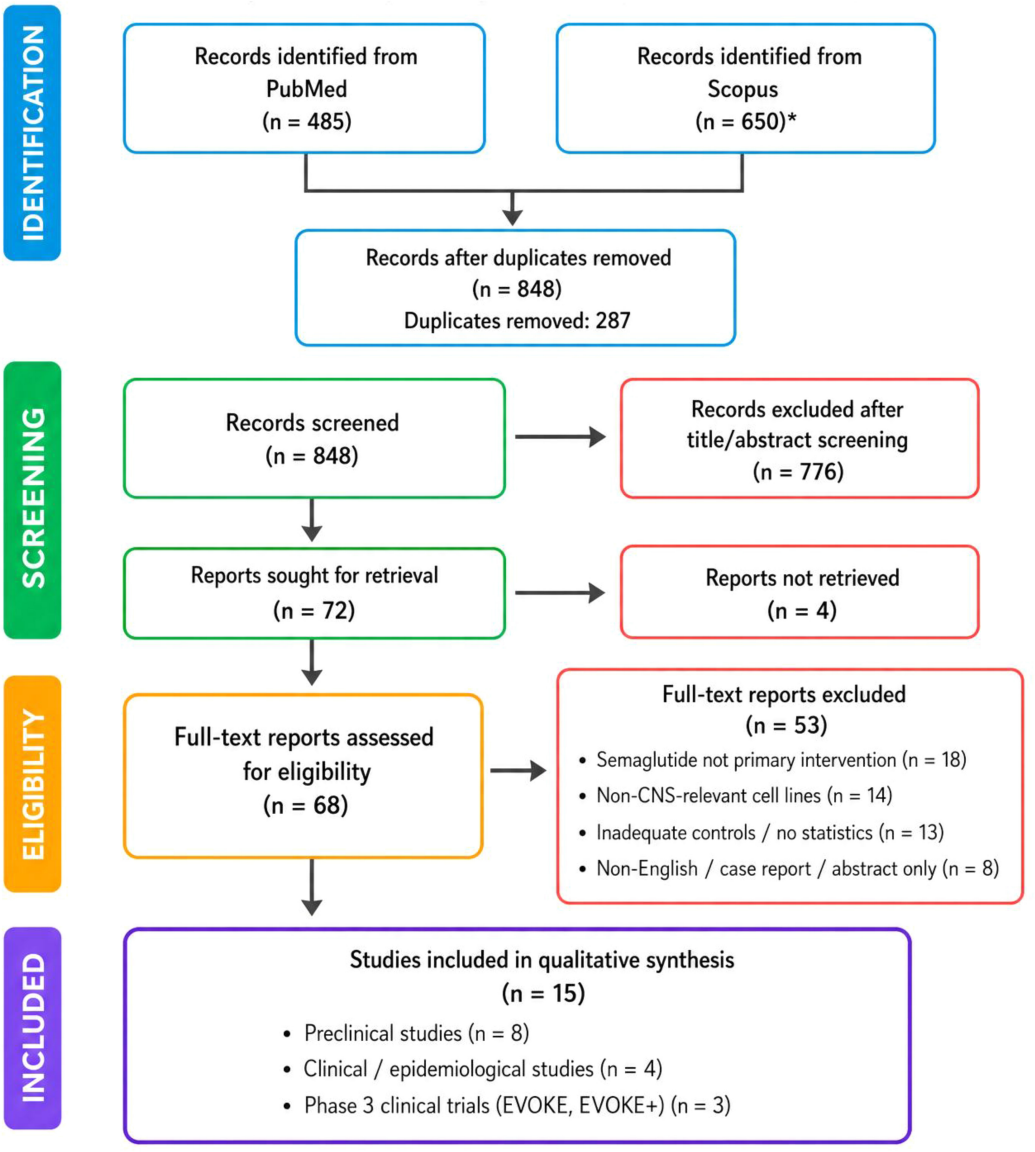
PRISMA flow diagram detailing the literature search, screening, and selection process. Explicit reasons for full-text exclusion are provided separately for preclinical studies and clinical/epidemiological studies.

To ensure a transparent and reproducible synthesis of the literature, a structured search strategy was employed in accordance with PRISMA guidelines. A systematic search of PubMed and Scopus databases was conducted for articles published between January 2018 and March 2025. The exact Boolean search string utilized was: (“semaglutide” OR “GLP-1 receptor agonist”) AND (“Alzheimer’s disease” OR “dementia” OR “cognitive dysfunction” OR “neuroprotection” OR “neuroinflammation” OR “clinical trial” OR “EVOKE”). To account for the pronounced heterogeneity of the preclinical evidence base, specific inclusion criteria for preclinical studies were defined as follows: (1) Model Type: utilization of established *in vivo* models (e.g., transgenic AD models like APP/PS1 or 3xTg-AD, neurotoxin-induced PD models, middle cerebral artery occlusion for ischemia, or high-fat diet for metabolic dysfunction) or relevant *in vitro* neuronal/glial cell lines; (2) Species: murine models (mice and rats) or human-derived cell lines; (3) Intervention Timing: clear delineation of preventive/co-treatment paradigms versus therapeutic paradigms; and (4) Outcome Measures: reporting of defined cognitive/behavioral tasks (e.g., Morris water maze) or specific neuropathological markers (e.g., Aβ plaque load, tau phosphorylation, inflammatory cytokines). For clinical and epidemiological evidence, inclusion criteria comprised: (1) large-scale observational studies reporting dementia incidence; and (2) randomized controlled trials reporting cognitive endpoints. Exclusion criteria applied across all domains were: non-English language publications, case reports, and conference abstracts without full-text data. Furthermore, during the full-text screening phase, additional exclusion criteria were applied to ensure focus and rigor: (1) studies where semaglutide was not the primary intervention or was only a minor comparator; (2) *in vitro* studies not utilizing CNS-relevant cell lines; and (3) studies lacking adequate control groups or failing to report statistical significance. Study screening was performed independently by two authors (A.H.A. and A.K.A.), with discrepancies resolved by consensus. The complete selection process, including the number of records identified, screened, and excluded with explicit reasons (separated for preclinical and clinical/epidemiological literature), is documented in the PRISMA flow diagram (Figure 1). Finally, studies published prior to 2018 were included only if they provided foundational pharmacokinetic or pharmacodynamic data on GLP-1 receptor distribution in the CNS or on the initial development of acylated GLP-1 analogs.

## 3 Pharmacology and central nervous system mechanisms

The therapeutic potential of semaglutide within the central nervous system (CNS) is intrinsically associated with the distribution and function of its target, the glucagon-like peptide-1 (GLP-1) receptor, as well as the drug’s distinctive pharmacological properties that influence its interaction with the brain. A comprehensive understanding of these factors is essential for interpreting the varied outcomes observed in clinical trials.

### 3.1 GLP-1 receptors in the brain

GLP-1 receptors are expressed in various regions of the brain, in addition to the pancreas and gut, highlighting the hormone’s function as a neuropeptide in the brain. Notable areas of expression include the brainstem, hypothalamus, hippocampus, and cortex (Cork et al., 2015; Graham et al., 2020). This distribution enables GLP-1 signaling to affect numerous central processes, such as appetite regulation, energy homeostasis, reward pathways, and learning and memory. The activation of these receptors in the hippocampus, a region crucial for memory formation, is believed to enhance synaptic plasticity and neurogenesis, providing a direct biological basis for the cognitive-enhancing effects observed in preclinical studies (During et al., 2003).

### 3.2 Semaglutide’s pharmacological profile and the blood-brain barrier

Semaglutide is a GLP-1 analog with approximately 94% homology to human GLP-1. Its distinctive characteristic is a structural modification involving the attachment of a C18 fatty diacid chain (acylation) via a spacer to the lysine at position 26. This modification facilitates strong, reversible binding to albumin, significantly extending its plasma half-life to approximately 1 week, thereby enabling weekly administration (Dhillon, 2018). This prolonged duration of action ensures sustained engagement of GLP-1 receptors, which is beneficial for metabolic regulation. However, this raises complex questions regarding its penetration and activity within the central nervous system (CNS).

A significant unresolved issue in the investigation of semaglutide for neurological disorders is its capacity to traverse the blood-brain barrier (BBB). The acylation process, which confers semaglutide with an extended half-life, also substantially increases its molecular size, a factor that is generally anticipated to impede its passage through the tightly regulated BBB. Indeed, initial preclinical studies employing radiolabeled tracers have indicated that acylated GLP-1 analogs, such as liraglutide and semaglutide, exhibit markedly limited brain penetration compared to their smaller non-acylated counterparts (Salameh et al., 2020). This observation led to the hypothesis that their neurological effects may be primarily mediated through peripheral mechanisms or indirect signaling pathways.

Recent evidence has elucidated the pharmacokinetics of semaglutide. Data from the EVOKE clinical trial program provided the first direct evidence in humans, indicating that although the concentration of semaglutide in the cerebrospinal fluid (CSF) is low, it is detectable. The CSF-to-plasma ratio is approximately 0.4%, confirming that a small but definite amount of the drug penetrates the central nervous system (CNS) (Scheltens et al., 2026). For context, the CSF exposure of monoclonal antibodies used in Alzheimer’s disease typically ranges from 0.1% to 0.3% of plasma concentrations, whereas small-molecule CNS drugs often exceed 5%, placing semaglutide within a low but pharmacologically plausible exposure range. This finding, while demonstrating limited penetration, aligns with the interpretation that semaglutide is not entirely excluded from the brain. It is hypothesized that semaglutide may enter through circumventricular organs, specialized brain regions such as the area postrema that lack a traditional blood-brain barrier, or via slow, low-capacity transport mechanisms (Athauda et al., 2026). This limited direct access has shifted the focus toward the significant role of indirect and peripheral mechanisms. Semaglutide’s potent systemic effects, including improved glycemic control, weight loss, and a marked reduction in systemic inflammation (as evidenced by an approximate 30% reduction in hs-CRP in the EVOKE trials), can substantially and positively impact brain health (Athauda et al., 2026; Lee et al., 2023). By reducing peripheral inflammatory signals, improving vascular health, and restoring metabolic homeostasis, semaglutide may alleviate the major drivers of neurodegeneration, suggesting that its neuroprotective benefits may not depend solely on high concentrations within the brain parenchyma.

## 4 Preclinical evidence: a foundation of neuroprotection

The considerable interest in semaglutide as a neurotherapeutic agent is derived from a robust body of preclinical evidence. In a diverse array of animal and cell-based models of neurological disorders, semaglutide has exhibited distinct protective effects, providing a biological rationale for its exploration in human studies. These investigations, summarized in Table 1, reveal a pleiotropic mechanism of action that extends significantly beyond its metabolic origins (Tipa et al., 2024).

**TABLE 1:** Summary of key preclinical studies on semaglutide in neurological models.

References	Model	Key cognitive & neuropathological findings	Proposed mechanism(s)	Intervention timing (preventive / therapeutic)	Key limitations
Wang et al., 2023	3xTg-AD Mouse (Alzheimer’s)	Improved learning and memory; decreased Aβ plaques and neurofibrillary tangles.	Activation of the GLP-1R/SIRT1/GLUT4 pathway improves glucose metabolism.	Preventive (treatment initiated before/during early pathology)	Transgenic model may not fully replicate human AD complexity; lacks late-stage therapeutic assessment.
Zhai et al., 2025	APP/PS1 Mouse (Alzheimer’s)	Enhanced cognitive function and attenuated Aβ accumulation.	Activation of AMPK and inhibition of the TLR4/NF-κB neuroinflammatory pathway.	Preventive (treatment initiated before severe plaque deposition)	Focuses primarily on amyloid pathology; findings may not translate to established, tau-dominant clinical disease.
Zhang et al., 2018, 2019	MPTP Mouse (Parkinson’s)	Improved motor impairment, protected dopaminergic neurons, and reduced α-synuclein levels.	Neuroprotection, anti-inflammatory effects, and superior potency to liraglutide.	Preventive / Early Therapeutic (co-administered with or shortly after toxin)	Toxin-induced model lacks the progressive, age-related neurodegenerative cascade of human Parkinson’s disease.
Basalay et al., 2019	Rat MCAO (Ischemic Stroke)	Reduced brain infarct size by 48%–63%; improved functional status.	Direct neuroprotection, independent of glucose-lowering effects.	Preventive (administered prior to ischemia-reperfusion)	Pre-ischemic administration does not reflect the typical clinical scenario of post-stroke treatment.
Tang et al., 2022	Mouse TMCAO (Ischemic Stroke)	Reduced infarct volume, improved neurobehavioral outcomes, and reduced BBB disruption.	Inhibition of neurotoxic A1 astrocyte conversion.	Therapeutic (administered shortly after reperfusion)	Short observation window; long-term neurofunctional recovery and survival were not fully evaluated.
Chen et al., 2023	High-Fat Diet Mouse (Metabolic)	Reversed cognitive deficits and improved spatial learning and memory.	Increased phosphorylation of key synaptic proteins (CACNA1D/A/B).	Preventive / Early Therapeutic (administered concurrently with high-fat diet)	Effects may be secondary to systemic metabolic improvement rather than direct central neuroprotection.
Sadek et al., 2023	EAE Mouse (Multiple Sclerosis)	Attenuated motor and cognitive dysfunction and reduced demyelination.	Activation of the PI3K/Akt/GSK-3β pathway; anti-inflammatory effects.	Preventive (administered from the day of immunization)	EAE model is primarily inflammatory and may not capture the progressive neurodegenerative phase of human MS.
Wang et al., 2021	PTZ Kindling Mouse (Epilepsy)	Reduced seizure severity, improved cognition, and prevented hippocampal neuron death.	Inhibition of NLRP3 inflammasome activation.	Preventive (administered prior to kindling)	Does not evaluate efficacy in reversing established chronic epilepsy or spontaneous recurrent seizures.

### 4.1 Alzheimer’s disease models

In transgenic mouse models that replicate key features of Alzheimer’s disease (AD) pathology, semaglutide has shown evidence of efficacy. In APP/PS1 and 3xTg-AD mice, treatment with semaglutide consistently resulted in improved performance in cognitive tasks, such as the Morris water maze, indicating enhanced spatial learning and memory. This functional improvement is accompanied by significant reductions in the core AD pathologies. Studies have documented decreased deposition of amyloid-beta (Aβ) plaques and lower levels of hyperphosphorylated tau, the two defining neuropathological hallmarks of this disease (Wang et al., 2023; Zhai et al., 2025). The mechanisms driving these effects are multifaceted, involving the suppression of neuroinflammation via the TLR4/NF-κB pathway and restoration of brain energy metabolism through the activation of the AMPK and SIRT1/GLUT4 signaling cascades (Wang et al., 2023; Zhai et al., 2025).

### 4.2 Parkinson’s disease models

Semaglutide has demonstrated significant neuroprotective potential in models of Parkinson’s disease (PD). In murine models treated with the neurotoxin MPTP, which selectively targets dopaminergic neurons to simulate PD, semaglutide administration effectively safeguarded these neurons from apoptosis, maintained dopamine concentrations, and subsequently enhanced motor function (Zhang et al., 2018). Additionally, semaglutide mitigated the accumulation of α-synuclein, the protein responsible for forming toxic Lewy body aggregates characteristic of PD (Zhang et al., 2019). Importantly, comparative studies have consistently shown that semaglutide surpasses its predecessor, liraglutide, in efficacy across most protective measures, likely because of its extended half-life and more sustained receptor engagement (Zhang et al., 2018, 2019).

### 4.3 Cerebrovascular disease models

Given the established association between vascular health and cognitive function, the effects of semaglutide on stroke models are of particular significance. In rodent models of acute ischemic stroke induced by middle cerebral artery occlusion (MCAO), semaglutide treatment resulted in a substantial reduction in the volume of the resultant brain infarct by 48%–63% and facilitated improved functional recovery (Basalay et al., 2019). The benefits of semaglutide in this context appear to be mediated not only by reducing inflammation and apoptosis but also by directly preserving the integrity of the blood-brain barrier (BBB). One study demonstrated that semaglutide inhibits the transformation of astrocytes into a neurotoxic A1 phenotype, a process that contributes to BBB breakdown and neuronal damage following ischemia (Tang et al., 2022).

### 4.4 Metabolic dysfunction and other models

Semaglutide’s capacity to ameliorate cognitive deficits induced by a high-fat diet in murine models underscores its potential to directly mitigate the neurological consequences associated with metabolic disorders (Chen et al., 2023). Furthermore, its significant anti-inflammatory properties have been demonstrated in epilepsy models, where it diminishes seizure severity by inhibiting the NLRP3 inflammasome (Wang et al., 2021), and in multiple sclerosis models, where it reduces demyelination and cognitive impairment (Sadek et al., 2023; Wang et al., 2023). These findings across various preclinical models suggest that the neuroprotective effects of semaglutide are not confined to a specific mechanism or disease context.

### 4.5 Translational limitations and contradictory evidence

Despite substantial neuroprotective evidence observed in numerous animal models, it is imperative to recognize significant translational limitations and some contradictory findings. A considerable portion of the preclinical literature involves the administration of semaglutide prior to or concurrently with disease induction, thereby reflecting a prevention or very early intervention paradigm rather than a treatment approach. This distinction is crucial when comparing these findings with clinical trials involving patients with established symptomatic disease. Additionally, variations in dosing equivalence, species-specific GLP-1 receptor expression, and metabolic responsiveness further complicate the direct translation of these findings to human diseases (De Giorgi et al., 2025).

Compounding this complexity, not all preclinical studies have yielded positive outcomes. A recent investigation by Forny Germano et al. (2024) reported that neither semaglutide nor the dual GIP/GLP-1 agonist tirzepatide influenced disease-related pathology, behavior, or cognitive function in 5XFAD and APP/PS1 mouse models of Alzheimer’s disease when administered after the establishment of pathology (Forny Germano et al., 2024). This finding highlights a fundamental distinction that must be applied when interpreting the preclinical literature as a whole: the majority of studies reporting positive outcomes employed a preventive or co-treatment paradigm, in which the drug was administered prior to or concurrently with pathological induction. Such designs are not representative of the clinical scenario, in which patients present with established, symptomatic disease. When intervention timing is shifted to a therapeutic paradigm — that is, treatment initiated after pathology is established — the evidence for efficacy becomes substantially weaker and less consistent. Furthermore, the inherent limitations of transgenic animal models must be acknowledged. These models replicate selected aspects of AD neuropathology (e.g., amyloid deposition) but fail to capture the full complexity of human disease, including its multifactorial etiology, heterogeneous progression, and the contribution of aging-related co-morbidities. Finally, the overwhelmingly positive preclinical literature is highly susceptible to publication bias, whereby neutral or negative findings are underreported. Furthermore, despite the application of rigorous inclusion and exclusion criteria in our methodology, it must be acknowledged that the systematic selection process itself may inadvertently introduce selection bias, potentially favoring studies with robust, positive findings that clearly meet all methodological thresholds. This combination of publication and selection bias may inflate the apparent magnitude and consistency of semaglutide’s neuroprotective effects in the preclinical literature. These considerations collectively underscore the critical importance of intervention timing and model selection, and suggest that the therapeutic window for semaglutide may close once significant pathology develops.

## 5 Molecular pathways of neuroprotection

**FIGURE 2:**
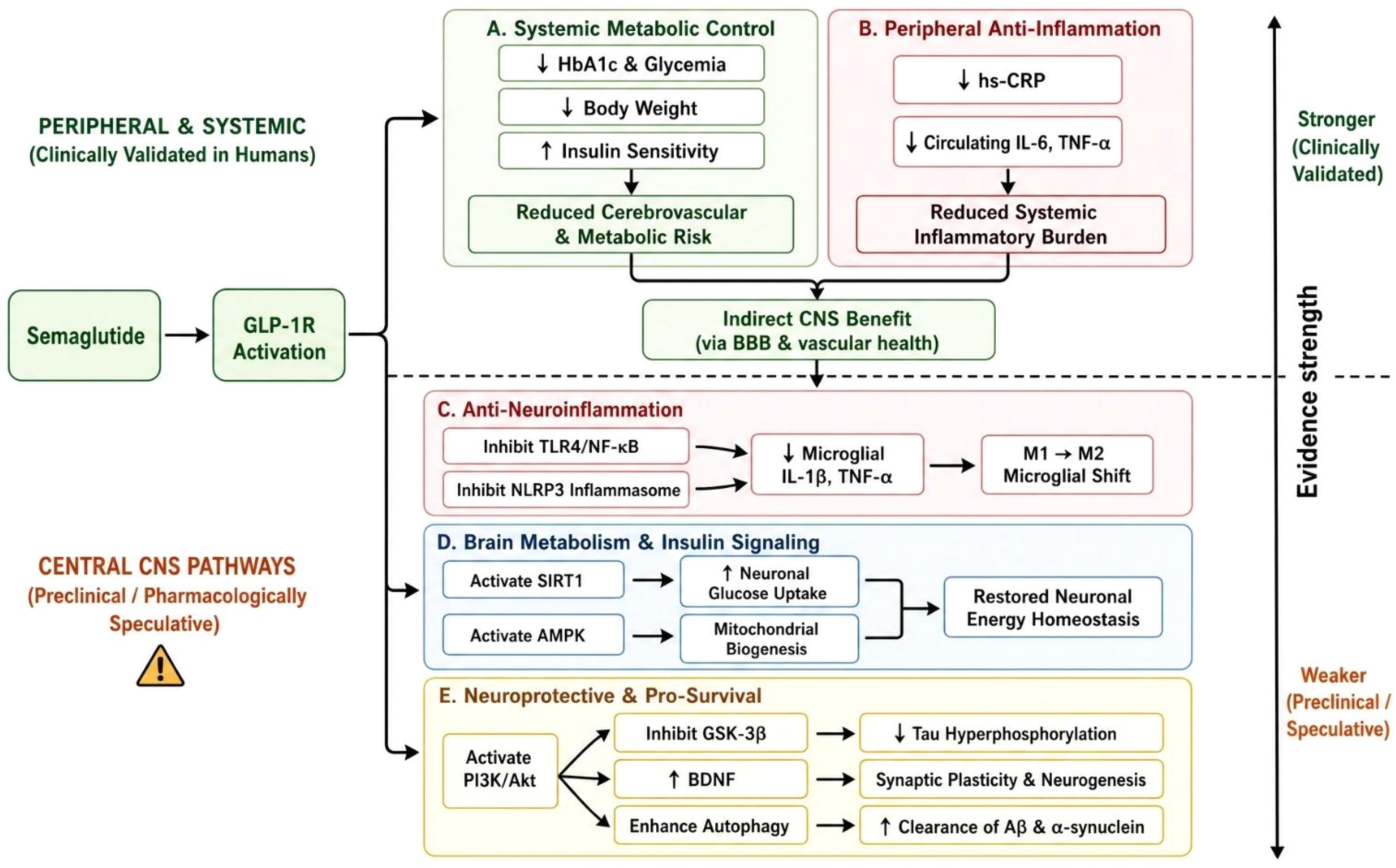
Molecular framework of semaglutide-mediated neuroprotection, stratified by translational evidence. GLP-1R activation engages two mechanistically and evidentially distinct tiers. The upper tier, established in randomized human trials, comprises systemic metabolic control (reduced glycemia and adiposity, improved insulin sensitivity) and peripheral immunomodulation (suppression of hs-CRP, IL-6, and TNF-α), which together attenuate cerebrovascular risk and preserve blood–brain barrier integrity. The lower tier, supported predominantly by rodent and *in vitro* studies, converges on three central modules: (i) microglial repolarization (M1?M2) via TLR4/NF-κB and NLRP3 inflammasome inhibition; (ii) SIRT1- and AMPK-dependent restoration of neuronal bioenergetics; and (iii) PI3K/Akt-driven pro-survival signaling that suppresses GSK-3β-mediated tau hyperphosphorylation, upregulates BDNF, and enhances autophagic clearance of Aβ and α-synuclein. Arrows indicate the direction of regulation. Central-tier pathways remain pharmacologically unverified at clinically attainable CNS exposures.

The principal molecular pathways involved in semaglutide-mediated neuroprotection are shown in Figure 2. These interconnected signaling cascades extend beyond mere GLP-1 receptor activation and impact inflammation, metabolism, and neuronal survival. However, it is essential to evaluate these mechanisms in the context of pharmacological feasibility. The pathways with the strongest human-relevant evidence are the peripheral anti-inflammatory effects (reduction in systemic hs-CRP and cytokines) and the systemic metabolic improvements (glycemic control, weight reduction), both of which are well-documented in clinical data. In contrast, direct central mechanisms — including PI3K/Akt-mediated neuroprotection, BDNF upregulation, and enhanced autophagy — are primarily supported by preclinical data obtained at drug concentrations substantially higher than those achievable in the human brain at therapeutic doses. These central pathways should therefore be regarded as biologically plausible but pharmacologically speculative in the clinical context, pending direct evidence of sufficient CNS target engagement. The varied neuroprotective effects of semaglutide observed in preclinical models are supported by its interaction with multiple interconnected intracellular signaling pathways. These mechanisms transcend simple GLP-1 receptor activation, affecting the fundamental processes of inflammation, metabolism, and cellular survival, as depicted in Figure 2.

### 5.1 Anti-neuroinflammatory effects

The principal mechanism by which semaglutide exerts its neuroprotective effects is through the suppression of neuroinflammation. Chronic inflammation, driven by the overactivation of glial cells, specifically microglia and astrocytes, is a common pathological characteristic of most neurodegenerative diseases. Semaglutide has been shown to directly counteract this process. Studies utilizing Alzheimer’s disease models suggest that semaglutide treatment inhibits the activation of microglia and astrocytes, thereby reducing the release of pro-inflammatory mediators (Zhai et al., 2025). This effect is mediated, at least in part, by the suppression of the Toll-like receptor 4 (TLR4)/nuclear factor-kappa B (NF-κB) signaling pathway (Zhai et al., 2025). Furthermore, evidence from epilepsy models indicates that semaglutide can inhibit the activation of the NLRP3 inflammasome, a multiprotein complex responsible for producing inflammatory cytokines such as IL-1β (Wang et al., 2021).

### 5.2 Enhanced brain metabolism and insulin sensitivity

The association between Alzheimer’s disease (AD) and metabolic dysfunction has led to the characterization of AD as “Type 3 diabetes,” a condition marked by impaired insulin signaling and glucose utilization in the brain (de la Monte and Wands, 2008). Semaglutide directly addresses this pathology by activating GLP-1 receptors in the brain, enhancing insulin sensitivity and improving cerebral glucose metabolism. Mechanistically, this is accomplished by activating key metabolic regulators. One significant pathway is the AMP-activated protein kinase (AMPK) system, which is the primary sensor of cellular energy status. Semaglutide appears to activate AMPK, which aids in restoring metabolic balance (Zhai et al., 2025). Another critical pathway involves sirtuin 1 (SIRT1), a protein that regulates cellular health and longevity. In AD mouse models, semaglutide increased the expression of SIRT1 and its downstream target, glucose transporter GLUT4, thereby enhancing glucose uptake and rectifying glycolytic deficits in the hippocampus (Athauda et al., 2026).

### 5.3 Direct neuroprotective and pro-survival pathways

In addition to its anti-inflammatory and metabolic effects, semaglutide activates pathways that directly enhance neuronal survival and resilience. It demonstrates significant anti-apoptotic properties by inhibiting programmed cell death in neurons subjected to toxic insults, such as Aβ or oxidative stress (Chang et al., 2020; Tipa et al., 2024). This is accomplished by modulating key apoptosis-regulating proteins, notably by increasing the expression of the pro-survival protein Bcl-2. Simultaneously, semaglutide enhances autophagy, a cellular mechanism responsible for the clearance of damaged organelles and misfolded protein aggregates. By augmenting autophagy, semaglutide facilitates the removal of toxic Aβ and α-synuclein, thereby preventing their accumulation and subsequent neurotoxicity (Chang et al., 2020). Moreover, activation of the GLP-1 receptor is known to increase the expression of Brain-Derived Neurotrophic Factor (BDNF), a crucial growth factor that supports neuronal survival, synaptic plasticity, and cognitive function (Roy et al., 2025).

### 5.4 Modulation of the gut-brain axis

Recent studies have underscored the significance of the gut-brain axis in maintaining neurological health. The gut microbiota composition influences neuroinflammation and brain function. Semaglutide, which acts on GLP-1 receptors in the gut, has the potential to modulate this microenvironment. Research conducted on obese mice has indicated that semaglutide treatment can reverse gut microbiota dysbiosis, particularly by increasing the abundance of beneficial bacteria, such as *Akkermansia muciniphila*. This enhancement in gut health is associated with improved cognitive performance and reduced systemic inflammation, suggesting that the neuroprotective effects of semaglutide are partially mediated by this complex axis (Feng et al., 2024).

Despite substantial mechanistic and preclinical evidence indicating neuroprotective effects, translating these findings into clinical benefits has proven challenging. The transition from experimental models to human diseases highlights a significant discrepancy between biological plausibility and therapeutic efficacy, necessitating a thorough examination of epidemiological and clinical trial evidence.

## 6 Clinical evidence: from epidemiological promise to trial disappointment

The progression of semaglutide from a metabolic medication to a prospective neurotherapeutic agent has been marked by a notable and challenging discrepancy between observational data and randomized controlled trial (RCTs) outcomes. While extensive epidemiological studies have consistently indicated a significant neuroprotective effect, the most conclusive clinical trials in Alzheimer’s disease have not corroborated this benefit, presenting a critical conundrum for the field.

### 6.1 Epidemiological and observational studies: a signal of prevention

Several extensive observational studies, primarily utilizing real-world data from nationwide health registries and insurance claims databases, have provided compelling evidence that the use of GLP-1 receptor agonists is associated with a reduced risk of developing dementia. These studies, summarized in Table 2, consistently demonstrate a protective effect, particularly in patients with type 2 Diabetes Mellitus (T2DM). One of the most influential studies, conducted by Wang et al. (2024), analyzed a substantial US real-world dataset and found that T2DM patients using semaglutide exhibited a 40%–70% lower risk of receiving a first-time Alzheimer’s disease (AD) diagnosis than those on other antidiabetic medications (Wang et al., 2024). Similarly, Lin et al. (2025) utilized the TriNetX database and found that semaglutide and the dual GLP-1/GIP agonist tirzepatide were associated with significantly lower risks of all-cause dementia, vascular dementia, and stroke. These findings are further supported by Chen et al. (2024) with the correct citation for a meta-analysis of GLP-1RA use and dementia risk. The reference currently listed for Chen et al. (2024) — a cardiovascular outcomes meta-analysis — does not support this statement and must be replaced with an appropriate dementia-focused meta-analysis (Chen et al., 2024; Hui et al., 2025).

**TABLE 2:** Summary of key epidemiological and observational studies on GLP-1 RAs and dementia risk.

References	Data source / population	Comparison	Key finding on dementia risk	Key limitations/confounders
Wang et al., 2024	US Nationwide Real-World Data (T2DM patients)	Semaglutide vs. other antidiabetics	40%–70% lower risk of first-time AD diagnosis with semaglutide.	Retrospective study; potential for indication bias and unmeasured confounders.
Lin et al., 2025	TriNetX Research Network (T2DM & Obesity)	Semaglutide/Tirzepatide vs. other antidiabetics	Significantly lower risk of dementia, including vascular dementia.	Real-world data; heterogeneity in patient populations and treatment adherence.
Nørgaard et al., 2022	Danish Health Registries & CV Outcome Trials	GLP-1 RA users vs. non-users	Users were less than half as likely to develop dementia as non-users.	Healthy-user bias; confounding by indication.
Hui et al., 2025	Meta-analysis of RCTs and observational studies	GLP-1 RAs vs. placebo/other drugs	Consistent reduction in future dementia risk with GLP-1 RAs.	Heterogeneity between studies with varying follow-up durations.

Although these findings are compelling, they must be interpreted with considerable caution, as observational studies are inherently limited in their ability to establish causality and are susceptible to multiple forms of bias. First, healthy-user bias is a major concern: individuals prescribed newer, more expensive agents such as semaglutide may systematically differ from comparator groups in terms of health behaviors, dietary patterns, physical activity, adherence to medical care, and access to healthcare resources. These differences, rather than the drug itself, could account for a portion of the observed risk reduction. Second, confounding by indication is highly probable, as treatment allocation is driven by underlying metabolic severity and clinical characteristics that are themselves associated with dementia risk. Third, immortal time bias may artificially inflate apparent protective effects if the period between cohort entry and treatment initiation is misclassified as exposed time. Fourth, residual confounding remains a fundamental limitation, as crucial variables — including socioeconomic status, educational attainment, baseline cognitive reserve, lifestyle factors, and genetic risk — are often inadequately captured or adjusted for in claims-based databases. Taken together, these methodological constraints mean that the robust and consistent epidemiological associations reported in this literature should be regarded as hypothesis-generating signals rather than definitive evidence of a causal neuroprotective effect. They provide a compelling rationale for prospective trials but cannot, in isolation, confirm that semaglutide prevents dementia.

### 6.2 The EVOKE and EVOKE+ trials: a negative verdict on treatment

The most comprehensive clinical investigation of semaglutide for Alzheimer’s disease (AD) was a Phase 3 program that included the EVOKE and EVOKE+ trials. These extensive, global, multi-year studies were designed to rigorously assess whether oral semaglutide could decelerate the progression of early stage symptomatic AD in over 3,800 participants (Scheltens et al., 2026). According to publicly disclosed topline results from the Phase 3 EVOKE and EVOKE+ programs — data that are currently available only as preliminary announcements and have not yet been fully peer-reviewed or published — the trials did not demonstrate clinical efficacy (Athauda et al., 2026). This preliminary nature must be acknowledged as a limitation of the current synthesis; the full dataset may reveal important subgroup effects or secondary findings not captured in topline summaries. Nevertheless, the absence of any signal on the primary endpoint across a large, well-powered trial is a robust negative finding (Cohen et al., 2022; Scheltens et al., 2026). The implications are significant: these results raise the possibility that semaglutide may have insufficient central target engagement at the doses used, that the pharmacodynamic effect on AD-relevant pathways may be inadequate, or that targeting metabolic and inflammatory pathways may represent a fundamental limitation when applied to established amyloid-driven Alzheimer’s disease.

However, the trials revealed some noteworthy changes in biomarkers. In a cerebrospinal fluid (CSF) substudy, participants receiving semaglutide exhibited modest yet statistically significant reductions (in the range of 10% or less) in several key biomarkers associated with Alzheimer’s disease (AD) pathology and neurodegeneration, including phosphorylated tau181 (p-tau181), phosphorylated tau217 (p-tau217), total tau, and the neuroinflammatory marker YKL-40 levels. Nevertheless, these alterations in CSF were not mirrored in plasma biomarkers and, more critically, did not result in any clinical benefit (Schauer et al., 2024; Teng et al., 2022). The trial outcomes clearly indicate that while semaglutide may engage with central AD pathology to a limited extent, it is not effective as a treatment for patients already diagnosed with the disease. It is essential to interpret the modest CSF biomarker changes with caution and to resist conflating biological activity with therapeutic efficacy. The well-documented disconnect between biomarker modulation and clinical outcomes in neurodegenerative diseases — illustrated most starkly by the history of anti-amyloid therapies — means that statistically significant reductions in p-tau or YKL-40 do not constitute evidence of meaningful clinical benefit. These findings demonstrate that semaglutide reaches the CNS and has some biological effect, but they do not support the conclusion that this effect is sufficient or appropriately targeted to alter disease progression in symptomatic AD.

### 6.3 Clinical trials in other cognitive domains

Beyond Alzheimer’s disease (AD), the cognitive effects of semaglutide have been investigated in various populations, yielding mixed outcomes. A Phase 2 randomized controlled trial (RCT) examining its impact on cognitive dysfunction in individuals with Major Depressive Disorder (MDD) reported no significant improvement in the primary outcome of executive functioning. However, secondary analyses suggested potential effects in other specific cognitive domains (Badulescu et al., 2026). Conversely, a study involving individuals with HIV indicated that semaglutide treatment was associated with enhanced performance in visuospatial abilities, language, and delayed recall, implying possible benefits for HIV-Associated Neurocognitive Disorders (HAND) (Atieh et al., 2025). These disparate findings suggest that the efficacy of semaglutide may be significantly influenced by the specific patient population and the underlying etiology of cognitive dysfunction.

## 7 Discussion

The investigation of semaglutide in the context of cognitive dysfunction serves as an illustrative example of translational medicine, marked by a notable discrepancy between preclinical potential, epidemiological indicators and conclusive clinical trial results. The primary question emerging from the extant evidence is: why did the pronounced neuroprotective effects observed in laboratory settings and the significant risk-reduction signals in population studies not translate into a therapeutic advantage for patients with early Alzheimer’s disease in the EVOKE trial? This discrepancy may be attributed to several alternative, non-mutually exclusive hypotheses that must be considered with equal weight: (1) a misalignment between the drug’s therapeutic window and the disease stage of the enrolled patients (the prevention hypothesis); (2) limited central nervous system penetration resulting in insufficient pharmacodynamic target engagement at the doses studied; (3) a fundamental mismatch between semaglutide’s primary metabolic and anti-inflammatory mechanisms and the amyloid- and tau-driven pathology that dominates established AD; and (4) the inherent limitations of the preclinical models from which the neuroprotective hypothesis was derived, which may not adequately predict efficacy in human disease. Each of these explanations carries distinct implications for future research directions and should not be dismissed in favor of a single unifying narrative.

### 7.1 Synthesizing the discrepancy: a prevention-dominant model

**FIGURE 3:**
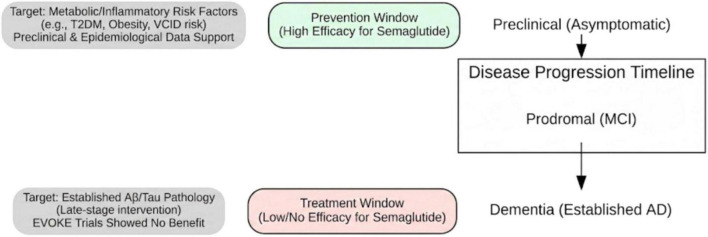
Prevention-treatment therapeutic window. This diagram illustrates that semaglutide’s efficacy is likely highest in the preclinical, asymptomatic phase of neurodegeneration, where it can target the upstream metabolic and inflammatory risk factors. In the later stages of dementia, where established amyloid and tau pathologies dominate, its therapeutic benefits diminish, as shown by the EVOKE trial outcomes. This diagram is a conceptual representation of a hypothesis and does not reflect direct clinical trial evidence.

A conceptual representation of the proposed therapeutic window within this prevention-focused framework is shown in Figure 3. The most plausible rationale for the discrepancy between preclinical/epidemiological success and clinical failure lies in the fundamental distinction between prevention and treatment. The preclinical models in which semaglutide demonstrates the most pronounced effects are typically models of prevention or very early intervention, wherein the drug is administered prior to or concurrently with pathological insult. Similarly, epidemiological studies have captured a preventive effect, wherein individuals taking semaglutide for metabolic reasons exhibit a reduced risk of developing dementia. In contrast, the EVOKE trials enrolled patients with established, amyloid-positive, early symptomatic Alzheimer’s disease (AD). These findings suggest that once AD pathology is established and cognitive symptoms emerge, the neurodegenerative cascade may be too advanced or may have become independent of the metabolic and inflammatory pathways that semaglutide effectively targets. The opportunity for intervention may have already passed, as shown in Figure 3.

This “prevention-dominant” model reinterprets the EVOKE trial outcomes not as a biological failure but as a failure to intervene at an optimal time. The modest yet statistically significant alterations in CSF biomarkers of inflammation and tau pathology indicate that semaglutide reaches the brain and exerts some biological effect. However, it is critical to distinguish between biological activity and therapeutic efficacy: these biomarker changes did not translate into any measurable clinical benefit, and should therefore be interpreted as evidence of limited central engagement rather than as support for the prevention-dominant hypothesis. However, this activity is insufficient to counteract established disease progression and yield clinical benefits.

### 7.2 Pathology mismatch: Alzheimer’s vs. vascular and metabolic cognitive impairment

A critical consideration is the potential incongruity between the pharmacological mechanism of a drug and the specific pathology of Alzheimer’s disease (AD). Although semaglutide exhibits pleiotropic effects, its most pronounced and direct actions are on the metabolic and inflammatory pathways. Epidemiological data consistently indicate the most substantial risk reduction for cognitive decline related to vascular and metabolic factors (Wang et al., 2025). This suggests that semaglutide may be significantly more effective in addressing the vascular and metabolic contributions to cognitive impairment and dementia (VCID) than the specific amyloid- and tau-driven pathology of AD. The EVOKE trials specifically selected an amyloid-positive population, which may not represent the cohort most likely to benefit from semaglutide’s primary mechanism. The drug’s lack of efficacy in this context does not preclude its potential utility in the broader, more heterogeneous population of older adults experiencing cognitive decline, where vascular and metabolic factors frequently play a major role.

### 7.3 Central vs. peripheral effects

The discourse surrounding the penetration of semaglutide across the blood-brain barrier (BBB) is pivotal to this study. The EVOKE trials confirmed a low but detectable concentration of semaglutide in the cerebrospinal fluid (CSF), thereby resolving the question of its entry into the central nervous system (CNS). This finding also underscores the probability that its most potent effects are mediated peripherally (Scheltens et al., 2026). The substantial reduction in systemic inflammation, as indicated by high-sensitivity C-reactive protein (hs-CRP) levels, alongside improvements in overall metabolic health, such as weight loss and glycemic control, represents significant systemic alterations that may indirectly benefit the brain by mitigating chronic inflammatory stimuli and enhancing cerebrovascular health. A critical and unresolved translational question is whether the ∼0.4% CSF-to-plasma ratio achieved at clinical doses is sufficient to meaningfully engage central GLP-1 receptors and activate the neuroprotective pathways described in Section 5. For context, the concentrations required to activate intracellular cascades such as PI3K/Akt, upregulate BDNF, or stimulate autophagy in neuronal tissue are derived from preclinical studies that used substantially higher relative exposures than those achievable in the human brain at therapeutic doses. The absence of clinical benefit in EVOKE is consistent with the interpretation that central pharmacodynamic effects are sub-therapeutic, and that the mechanistic pathways proposed on the basis of preclinical data may not be meaningfully engaged *in vivo* in humans. This represents a fundamental translational gap that must be acknowledged when interpreting the mechanistic evidence presented in this review. It is plausible that these indirect peripheral effects primarily drive the observed reduction in dementia risk in population studies. Nonetheless, these effects may be inadequate to reverse or arrest the progression of established, self-propagating intracerebral Alzheimer’s disease (AD) pathology, further supporting the prevention-dominant model.

### 7.4 Future directions

While current evidence does not substantiate the use of semaglutide as a treatment for established Alzheimer’s disease (AD), it suggests several promising avenues for future research.

#### 7.4.1 Prevention trials

The most promising potential of semaglutide and other GLP-1 receptor agonists lies in primary and secondary prevention. Future trials should be structured to assess whether initiating treatment in high-risk, cognitively unimpaired individuals (e.g., those with type 2 diabetes mellitus, obesity, and/or specific genetic risk factors such as APOE4) can delay or prevent cognitive decline onset. Such trials would represent the definitive test of the prevention-dominant hypothesis advanced in this review, and their results — whether positive or negative — would substantially clarify the role of GLP-1 receptor agonists in cognitive health.

#### 7.4.2 Vascular cognitive impairment

Given the strong indication of reduced risk of vascular dementia, dedicated trials are necessary to evaluate semaglutide in patients with established vascular cognitive impairment or those at high risk. This population is more likely to benefit from the potent metabolic and anti-inflammatory effects of the drug.

#### 7.4.3 Combination therapies

The modest biomarker effects observed in the EVOKE trials suggest that semaglutide interacts with central pathologic processes. This raises the possibility of synergistic effects when combined with drugs that directly target the core Alzheimer’s disease pathology, such as anti-amyloid monoclonal antibodies. A combination approach could address both upstream metabolic drivers and downstream pathological consequences.

#### 7.4.4 Next-generation agonists

The development of dual GLP-1/GIP receptor agonists (e.g., tirzepatide) and novel blood-brain barrier-penetrant formulations may offer superior neuroprotective efficacy by more potently engaging central receptors or providing a broader spectrum of action (Ghaleb et al., 2025).

### 7.5 Limitations of current evidence

Several limitations must be considered when interpreting the current evidence. First, a significant portion of the mechanistic support is derived from preclinical studies that utilize preventive or early intervention paradigms, which may not accurately reflect clinical treatment scenarios. Second, epidemiological studies indicating a reduced risk of dementia are susceptible to residual confounding factors, such as healthy user bias and treatment selection effects. Third, the heterogeneity in cognitive endpoints across clinical investigations hinders direct comparisons between studies. Finally, there remains uncertainty regarding the relative contributions of direct central nervous system exposure versus indirect peripheral metabolic effects in mediating the neurological actions of semaglutide. These limitations highlight the necessity for carefully designed future trials targeting appropriate disease stages and patient populations.

## 8 Conclusion

The semaglutide case exemplifies the intricate nature of neurodegenerative diseases and the challenges inherent in therapeutic development. It demonstrates potent and multifaceted neuroprotective properties across a diverse range of preclinical models through a combination of anti-inflammatory, metabolic, and pro-survival pathways. This preclinical promise is robustly supported by extensive epidemiological data that consistently link its use to a significantly reduced risk of incident dementia. However, this potential has not been realized in the treatment of established Alzheimer’s disease, as evidenced by the negative outcomes of the pivotal phase 3 EVOKE clinical trial program. Collectively, this evidence is consistent with the hypothesis that semaglutide’s primary value in the context of cognitive health may lie in prevention rather than treatment, particularly for cognitive decline driven by vascular and metabolic dysfunctions. However, this prevention-dominant framework remains speculative. No randomized controlled trial has yet demonstrated that semaglutide prevents cognitive decline or dementia incidence. Rigorous, long-term prospective trials in asymptomatic, high-risk populations — such as individuals with type 2 diabetes, obesity, or APOE4 carrier status — are required to definitively validate or refute this hypothesis. The progression of semaglutide from a metabolic drug to a potential neuroprotective agent is ongoing, but its future role in neurology will likely focus on the prevention of neurodegeneration rather than the treatment of established diseases.
